# Susceptibility of Blood Orange Cultivars to Chilling Injury Based on Antioxidant System and Physiological and Biochemical Responses at Different Storage Temperatures

**DOI:** 10.3390/foods9111609

**Published:** 2020-11-05

**Authors:** Fariborz Habibi, Asghar Ramezanian, Fabián Guillén, Domingo Martínez-Romero, María Serrano, Daniel Valero

**Affiliations:** 1 Department of Horticultural Science, School of Agriculture, Shiraz University, Shiraz 71441-65186, Iran; fariborz_h659@yahoo.com (F.H.); ramezanian@shirazu.ac.ir (A.R.); 2 Department of Food Technology, University Miguel Hernández, Ctra. Beniel km. 3.2, Orihuela, 03312 Alicante, Spain; fabian.guillen@umh.es (F.G.); dmromero@umh.es (D.M.-R.); 3 Department of Applied Biology, University Miguel Hernández, Ctra. Beniel km. 3.2, Orihuela, 03312 Alicante, Spain; m.serrano@umh.es

**Keywords:** antioxidant enzymes, hydrogen peroxide, phenylalanine ammonia-lyase, proline, scanning electron microscopy

## Abstract

Susceptibility of four blood orange cultivars (‘Moro’, ‘Tarocco’, ‘Sanguinello’ and ‘Sanguine’) to chilling injury (CI) was studied. Antioxidant enzymes as well as physiological and biochemical changes were measured monthly at 2 and 5 °C plus 2 days at 20 °C for shelf life. At 2 °C, CI symptoms were higher than at 5 °C, and ‘Moro’ and ‘Tarocco’ had significantly higher CI than ‘Sanguinello’ and ‘Sanguine’. ‘Moro’ and ‘Tarocco’ had the highest electrolyte leakage, malondialdehyde, hydrogen peroxide (H_2_O_2_) and polyphenol oxidase activity and lower phenylalanine ammonia-lyase compared with ‘Sanguinello’ and ‘Sanguine’. The scanning electron microscopy (SEM) micrographs revealed that ‘Moro’ and ‘Tarocco’ showed severe fractures in the flavedo due to CI. ‘Sanguinello’ and ‘Sanguine’ were more tolerant to CI due to an increase of catalase, ascorbate peroxidase and superoxide dismutase, which could prevent the loss of membrane integrity and alleviate CI symptoms. Hierarchical clustering analysis (HCA) for cultivars and temperatures revealed four main clusters. The first cluster included ‘Moro’ and ‘Tarocco’ at 2 °C, and the second cluster included ‘Moro’ and ‘Tarocco’ at 5 °C. The third cluster involved ‘Sanguinello’ and ‘Sanguine’ at 2 °C, and the fourth cluster included ‘Sanguinello’ and ‘Sanguine’ at 5 °C. The order of susceptibility of cultivars to CI was ‘Moro’ > ‘Tarocco’ > ‘Sanguine’ > ‘Sanguinello’.

## 1. Introduction

Blood oranges belong to sweet orange species (*Citrus sinensis* L. Osbeck) and are recognized among the oranges due to the presence of anthocyanins in the flesh and sometimes in the fruit peel [1]. ‘Moro’, ‘Tarocco’, ‘Sanguinello’ and ‘Sanguine’ are the most important commercial cultivars of blood oranges [2] and they arose from a spontaneous bud mutation [3]. Blood oranges have gained popularity among consumers due to their content of bioactive compounds such as anthocyanins, ascorbic acid, hydroxycinnamic acids and flavonoids [4]. Distinct pomological characteristics of these cultivars are fruit size, rind thickness and anthocyanin pigmentation in the flesh and peel [5]. For example, ‘Moro’ is medium size and has a sweet flavour with a deep red juice colour and bright red blush rind. ‘Tarocco’ is medium to large fruit size, with a low red juice colour, sweetness and juiciness, and has slightly blushed rind. ‘Sanguinello’ is smaller than the other fruits, less round but rather oval to egg-shaped, with few or no seeds, reddish compact peel and sweet and tender flesh. ‘Sanguine’ is a thin peel cultivar, having good juice colour and being easy to peel [5]. 

Chilling stress can induce oxidative damage in citrus fruit. Oxidative damage results in the generation of reactive oxygen species (ROS) and is thought to be a response of sensitive fruit to chilling temperatures that can affect cell membrane integrity [6]. In these conditions, enzymatic and non-enzymatic antioxidant systems can scavenge the ROS, whereas proline synthesis can maintain the cellular membrane integrity by osmoregulation and increase the resistance of fruit to chilling temperature [7].

Blood oranges exhibit some responses at the cellular level after exposure to cold stress. The common responses include changes in cell structure, fatty acid saturation index, lipid peroxidation, electrolyte leakage (EL), proline content, hydrogen peroxide (H_2_O_2_), malondialdehyde (MDA), activity of antioxidant enzymes and epidermis structure, among others [8]. In addition, chilling stress can change the cellular metabolism and reduce cell energy status, leading to chilling injury (CI) symptoms such as scalding, rind pitting and necrotic areas, watery breakdown and browning [8,9]. Postharvest treatment by hot water dipping (HWD, 3 min at 50 °C) and hot air treatment (HAT, 37 °C for 48 h) on blood orange fruit during cold quarantine at 1 °C for 16 days, showed that HWD and HAT similarly reduced CI in all cultivars, and neither treatment caused visible damage to the fruit [10]. In addition, vacuum infiltration of putrescine alleviated CI and maintained fruit quality of two blood orange cultivars (‘Moro’ and ‘Tarocco’) during cold storage [1]. It has been reported that enhancing chilling tolerance in blood orange fruit exposed to chilling stress depended on inducing antioxidant enzymes, increasing phenylalanine ammonia-lyase (PAL) activity and promoting the proline content and subsequently achieving increased maintenance of membrane integrity [8].

Blood oranges are sensitive to cold stress and exhibit CI symptoms when stored at 5 °C, depending on cultivars and storage temperatures [8]. However, storage of blood orange at cold temperatures is necessary for extending their postharvest life [1]. In recent years, some postharvest treatments such as γ-aminobutyric acid (GABA), methyl jasmonate (MeJA) and methyl salicylate (MeSA) have been applied for increasing chilling tolerance of blood oranges as well as controlling the peel’s physiological disorders [8]. Blood oranges have been stored up to 4 and 5 months at 8 and 3 °C, respectively, although depending on the cultivars [11]. To date, no information is available on physiological and biochemical responses of blood orange cultivars during long-term storage at 2 and 5 °C. Physiological and biochemical studies may provide very useful information about the involved mechanisms for chilling tolerance of blood orange cultivars in response to cold stress and how these responses are different among cultivars. Therefore, the objective of this study was to assess the susceptibility of blood orange cultivars, namely ‘Moro’, ‘Tarocco’, ‘Sanguinello’ and ‘Sanguine’, to CI when stored at 2 and 5 °C (chilling temperatures). In addition, the physiological and biochemical responses occurring during 180 days of storage and the role of the antioxidant system in increasing CI tolerance were evaluated. Furthermore, scanning electron microscopy (SEM) micrographs were captured to survey the epidermis structure of each cultivar at both temperatures to confirm the chilling tolerance of blood orange fruit.

## 2. Materials and Methods

### 2.1. Plant Material and Storage Conditions

Blood orange cultivars, i.e., ‘Moro’, ‘Tarocco’, ‘Sanguinello’ and ‘Sanguine’, were harvested in mid-January 2018 from a commercial orchard at Dashtenaz Company, Sari, Mazandaran, Iran (latitude 36°33′47.95″ N, longitude 53°03′36.32″ E). Trees were seven years old and grafted on ‘C-35′ citrange (*Citrus sinensis* L. Osbeck × *Poncirus trifoliata* L. Raf.) rootstock and planted in a loamy soil, at 7 × 5 m, with drip irrigation system. Trees were irrigated based on their water requirements during growth cycle by using a programmer drip irrigation system consisting of two drip irrigation lines per row with three emitters per tree. All trees were grown under the same conditions and cultural practices. In this area of Iran, the climate is subtropical and the four cultivars reach full maturity in January, and they were harvested at the same time and according to total soluble solids (TSSs) and titratable acidity (TA) ratio. Blood orange cultivars were immediately transported to the postharvest laboratory and selected based on uniformity of size and checked for no defects or rind injuries. Pomological data for each cultivar (mean data of 3 replicates of 5 fruit) were TSS (‘Moro’ = 10.3 ± 0.37, ‘Tarocco’ = 11.26 ± 0.28, ‘Sanguinello’ = 10.7 ± 0.44 and ‘Sanguine’ = 12.13 ± 0.47), TA (‘Moro’ = 1.49 ± 0.05, ‘Tarocco’ = 1.32 ± 0.08, ‘Sanguinello’ = 1.95 ± 0.08 and ‘Sanguine’ = 1.67 ± 0.05) and peel colour in ‘Moro’ (L * = 72.21 ± 0.3, a * = 27.41 ± 0.48, b * = 69.75 ± 1.82), ‘Tarocco’ (L * = 71.77 ± 0.53, a * = 28.18 ± 0.92, b * = 73.09 ± 0.54), ‘Sanguinello’ (L * = 69.92 ± 0.44, a * = 24.94 ± 1.31, b * = 70.22 ± 1.27) and ‘Sanguine’ (L * = 72.67 ± 0.35, a * = 22.3 ± 0.85, b * = 70.93 ± 0.88). Fruit were disinfected with 2% sodium hypochlorite (NaOCl) solution for 5 min and then rinsed with distilled water. The number of fruits for each cultivar was 210 for 7 sampling times for both temperatures (105 fruit for 2 °C and 105 fruit for 5 °C). Five fruit were placed in each polyethylene bag. For each cultivar and temperature after 0, 30, 60, 90, 120, 150 and 180 days of cold storage, 5 fruit from each replicate were transferred 2 days at 20 °C. Fruit were divided into sets of three replicates and placed in polyethylene bags (25 × 35 cm) with 16 holes (2 mm diameter) and separately stored for up to 180 days at 2 and 5 °C and 90% relative humidity (RH) for the following analytical determinations in the flavedo tissue: chilling injury (CI) index, electrolyte leakage (EL), MDA content, proline content, hydrogen peroxide (H_2_O_2_) content, catalase (CAT), peroxidase (POD), ascorbate peroxidase (APX), superoxide dismutase (SOD), phenylalanine ammonia-lyase (PAL) and polyphenol oxidase (PPO) activities and total protein content. Scanning electron microscopy (SEM) micrographs of flavedo tissue were captured at the end storage.

### 2.2. CI Index Evaluation

CI symptoms were determined by evaluation of rind pitting and necrotic areas of the cultivars during cold storage. Five fruit from the three replicates were visually evaluated on a four-point scale as 0 (no injury), 1 (slight injury), 2 (medium injury) and 3 (severe injury). CI index was measured using the following Equation (1) [12]:

(1) $$CI\;index=\;\frac{\Sigma \;\left(CI\;level\right)\;\times \;\left(Number\;of\;fruit\;at\;CI\;level\right)}{Total\;number\;of\;fruit\;in\;the\;tretament}$$

### 2.3. Peel Thickness Determination and Electrolyte Leakage (EL)

Peel thickness was measured in recently harvested blood oranges (15 fruit) with a digital calliper, and results (mean ± SE, standard error) were expressed in mm. For EL evaluation, ten discs of flavedo tissue were excised using a cork borer with a 0.5 cm diameter and rinsed twice with distilled water. The discs were inserted in a 15 mL falcon tube containing 10 mL of 0.3 mol L^−1^ mannitol solution and shaken at room temperature for 3 h. First conductivity (EC1) was measured after 3 h of shaking, and second (EC2) after boiling in water at 100 °C for 5 min and cooling down at room temperature, using a conductivity meter (AZ-86505, Taichung, Taiwan). The percentage of electrolyte leakage was calculated using the following Equation (2) [7]:

(2) $$EL\left(\%\right)=\;100\;\times \;\frac{EC1}{EC2}$$

### 2.4. MDA Content

To evaluate the MDA content, 0.1 g of flavedo tissue were homogenized in 2 mL of 1% trichloroacetic acid (TCA) and centrifuged at 10,000× *g* for 10 min. Supernatant (250 µL) was mixed with 1 mL of 20% TCA containing 0.5% thiobarbituric acid (TBA) and the mix was held in hot water at 90 °C for 30 min, then immediately cooled in an ice bath and centrifuged. Absorbance was read at 532, 600 and 450 nm by a microplate reader spectrophotometer (Epoch Biotek, Winooski, VT, USA). MDA was calculated as indicated by Wang et al. [7], and results (mean ± SE) expressed as µmol kg^−1^.

### 2.5. H_2_O_2_ Content

Flavedo (0.2 g) was homogenized in 2 mL of 1% (*w/v*) trichloroacetic acid (TCA) and centrifuged at 4 °C for 10 min at 10,000× *g*. The supernatant (250 µL) was then mixed with 250 µL of 100 mmol L^−1^ phosphate buffer (pH = 7) and 500 µL of 1 mol L^−1^ of potassium iodide (KI) in a 2 mL Eppendorf tube. Then, the mixture solution was vortexed and its absorbance was read at 390 nm using a microplate reader spectrophotometer (Epoch Biotek, Winooski, VT, USA). The content of H_2_O_2_ was determined with a standard curve of H_2_O_2_, and results (mean ± SE) were expressed as mmol kg^−1^ fresh weight [13].

### 2.6. Proline Content

To evaluate the proline content, 0.5 g of flavedo were homogenized in 10 mL of 3% (*v/v*) sulfosalicylic acid and then centrifuged at 12,000× *g* for 10 min. Two mL of the supernatant were mixed with 2 mL of glacial acetic acid and 2 mL of ninhydrin reagent in the test tube and held in hot water at 100 °C for 1 h. Solution was cooled immediately in an ice bath and 4 mL of toluene were added to the reaction mixture, which was vortexed for 20 s. The absorbance was measured at 520 nm by spectrophotometer (Dynamica, Livingston, UK). The proline content was determined with a standard curve of proline, and results (mean ± SE) expressed as g kg^−1^ fresh weight [14].

### 2.7. Enzyme Activity Determination

For the enzyme extraction (CAT, APX, SOD and POD activities), 0.5 g of flavedo tissue were excised and ground in liquid nitrogen with a mortar and pestle, then homogenized with 2 mL of extraction buffer (50 mmol L^−1^ potassium phosphate buffer, pH = 7, containing 2 mmol L^−1^ ethylene diamine tetraacetic acid and 1% polyvinylpyrrolidone) in a 2 mL Eppendorf tube and centrifuged at 13,000× *g* for 10 min at 4 °C. The supernatant was taken for measurement of all enzyme activities and total protein content [15]. All enzyme activities were measured by spectrophotometry (Dynamica, Livingston, UK) and the specific activity was expressed as U mg^−1^ protein. CAT activity was measured by the H_2_O_2_ decomposition after 1 min of reaction at 240 nm [16]. APX activity was measured according to Nakano and Asada [17] by measuring the absorbance at 290 nm. SOD activity was measured according to the Beauchamp and Fridovich [18] method by measuring the absorbance at 560 nm. POD activity was measured using guaiacol as substrate, and after 2 min of reaction the absorbance was measured at 470 nm [16].

The PAL activity was determined by measuring the absorbance of trans-cinnamic acid at 290 nm [19]. For enzyme extraction, 0.5 g of fruit peel were excised and ground in liquid nitrogen with a mortar and pestle, then 2 mL of extraction buffer were added (containing 100 mmol L^−1^ of sodium borate buffer (pH = 7), 5 mmol L^−1^ β-mercaptoethanol and 1% PVP) and centrifuged at 13,000× *g* during 20 min at 4 °C. The activity of PPO was determined using the Silva and Koblitz [20] method at 425 nm. For enzyme extraction, 0.2 g of fruit peel were excised and ground in liquid nitrogen with a mortar and pestle, then mixed with 2 mL of extraction buffer (containing 100 mmol L^−1^ of potassium phosphate buffer (pH = 7.8) and 1% PVP) and centrifuged at 13,000× g for 15 min at 4 °C. Results for all enzymes were expressed in U mg^−1^ protein.

### 2.8. Scanning Electron Microscopy (SEM) Micrographs 

SEM micrographs were captured to study the epidermis structure of the peel at both storage temperatures. For preparing SEM images, flavedo tissue (1 × 1 cm) from each cultivar was excised with a scalpel blade from the fruit equatorial area. Samples were lyophilized using a freeze-dryer (FD-5003-BT, manufacturer, Tehran, Iran) and attached on a stub, and the peel surface was coated with a thin layer of gold (Desk Sputter Coater Dsr1, Nanostructural Coatings, Tehran, Iran). Digital SEM images were taken using a scanning electron microscope (Scanning Electron Microscope, TESCAN vega3, Brno, Czech Republic) at 500× magnification [21].

### 2.9. Statistical Analyses

The experiment was conducted according to a completely randomized design (CRD) with three replicates. Data were analysed using three-factor (4 cultivars, 2 temperatures and 7 storage times) analysis of variance (ANOVA). Mean comparisons were done by least significant difference (LSD) test (*p* < 0.05) with standard errors of means. Data analyses were performed with SAS software package v. 9.4 for Windows (SAS Institute, North Carolina State University, Cary, NC, USA). Hierarchical cluster analysis (HCA) was conducted using a Ward linkage method with Euclidean distance, and the data were depicted in a dendrogram plot by Minitab v. 16 software (Pennsylvania State University, State College, PA, USA). Correlation between peel thickness and CI at both temperatures was performed with SigmaPlot v. 11 software (Systat Software Inc., San Jose, CA, USA).

## 3. Results

### 3.1. CI Index, Peel Thickness, EL and SEM Micrograph

**Figure 1:**
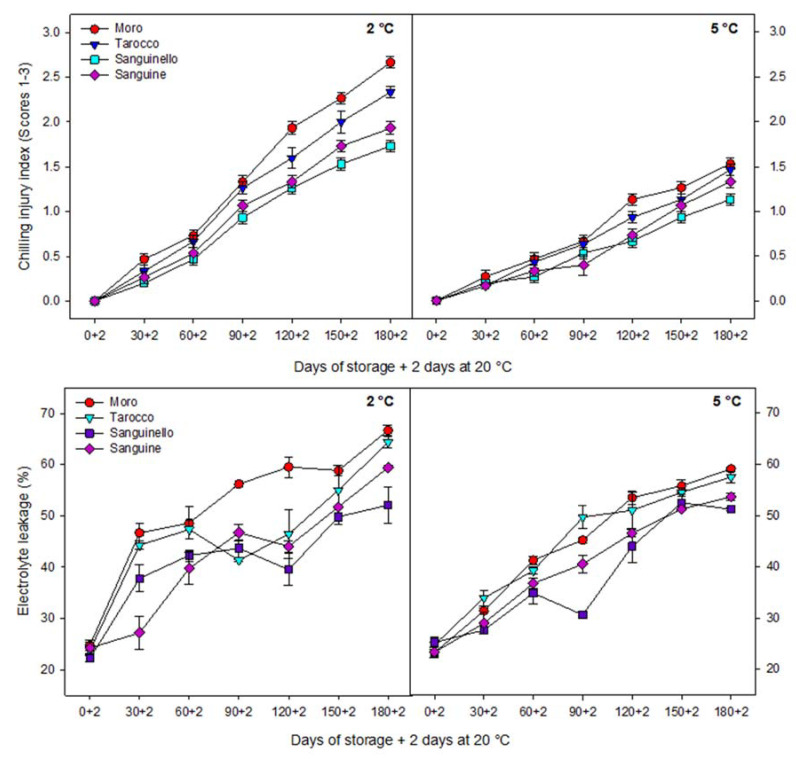
Chilling injury (CI) index and electrolyte leakage (EL) of blood orange cultivars (‘Moro’, ‘Tarocco’, ‘Sanguinello’ and ‘Sanguine’) during 180 days of cold storage at 2 and 5 °C and 90% relative humidity (RH) and 2 days at 20 °C. Vertical bars represent ± standard error (SE) of means. Least significant difference (LSD) (*p* < 0.05) value is 0.18 for CI and 5.17 for EL.

CI index increased during storage for all cultivars and for both temperatures (Figure 1). CI symptoms appeared after 30 days of cold storage at both temperatures although, as expected, CI was significantly higher (42% more, on average) at 2 °C than at 5 °C for all blood oranges along the 180 days of storage. Among cultivars, ‘Moro’ and ‘Tarocco’ showed significantly higher CI symptoms than ‘Sanguine’ and ‘Sanguinello’, the latter having the lowest CI.

The cultivars differed in peel thickness, with ‘Tarocco’ being the thickest (5.01 ± 0.34 mm), followed by ‘Moro’ (4.52 ± 0.11 mm), ‘Sanguinello’ (3.42 ± 0.09 mm) and ‘Sanguine’ (2.93 ± 0.08 mm). Membrane permeability of blood orange cultivars was evaluated by measuring peel EL. As shown in Figure 1, EL significantly increased for all cultivars with a similar trend during 180 days of storage at both temperatures, although EL at 2 °C was greater than at 5 °C. ‘Moro’ had higher EL (66.71% ± 1.02%) than ‘Tarocco’, ‘Sanguinello’ and ‘Sanguine’. Thus, at the end of storage, the lowest EL was observed in ‘Sanguinello’ cultivar at both temperatures, 52.07% ± 3.46% and 51.20% ± 0.32%, respectively. EL in ‘Moro’ was 3.5%, 21.93% and 10.92% higher than ‘Tarocco’, ‘Sanguinello’ and ‘Sanguine’ cultivars, respectively, at the last sampling date at 2 °C.

**Figure 2:**
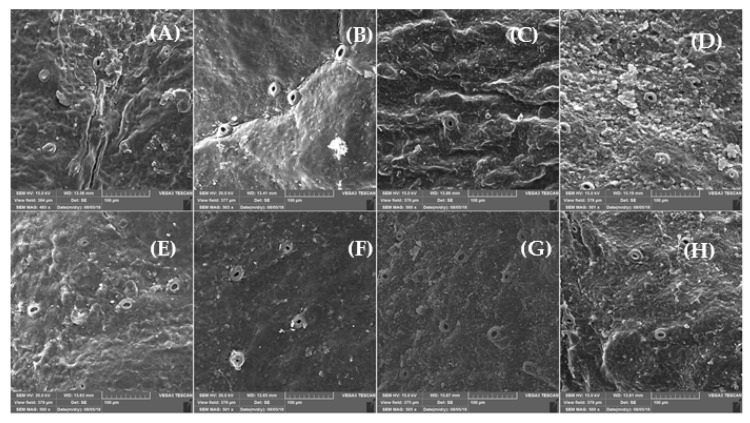
Scanning electron micrographs (500× magnification) of the flavedo of blood orange cultivars after 180 days of cold storage plus 2 days at 20 °C; (**A**) ‘Moro’ at 2 °C; (**B**) ‘Tarocco’ at 2 °C; (**C**) ‘Sanguinello’ at 2 °C; (**D**) ‘Sanguine’ at 2 °C; (**E**) ‘Moro’ at 5 °C; (**F**) ‘Tarocco’ at 5 °C; (**G**) ‘Sanguinello’ at 5 °C; (**H**) ‘Sanguine’ at 5 °C.

SEM micrographs were captured to study the epidermis structure of the peel at the 2 storage temperatures (Figure 2A–H), in which fractures and cuticular ridges at 2 °C were greater than at 5 °C for all cultivars. ‘Moro’ and ‘Tarocco’ showed a rough structure with severe fractures and cuticular ridges of the peel in comparison with ‘Sanguinello’ and ‘Sanguine’ at 2 °C (Figure 2A–D). Moreover, visible fractures in ‘Sanguinello’ were less pronounced than ‘Sanguine’ (Figure 2C,D).

The micrographs also showed that epicuticular wax crystals were lost and the remaining wax layer acquired an irregular morphology due to CI at 2 °C. Cultivars stored at 5 °C had an intact peel surface, without the appearance of severe fractures, but epicuticular wax was also lost for all blood oranges (Figure 2E–H). Among cultivars, ‘Moro’ and ‘Tarocco’ had lower epicuticular wax and more granular surface in comparison with ‘Sanguinello’ and ‘Sanguine’ cultivars (Figure 2E,F). Interestingly, ‘Sanguinello’ had a fine structure of epicuticular wax, with no wrinkling signs and lower granule surface in comparison with ‘Sanguine’ (Figure 2G,H).

### 3.2. H_2_O_2_, MDA and Proline Content

**Figure 3:**
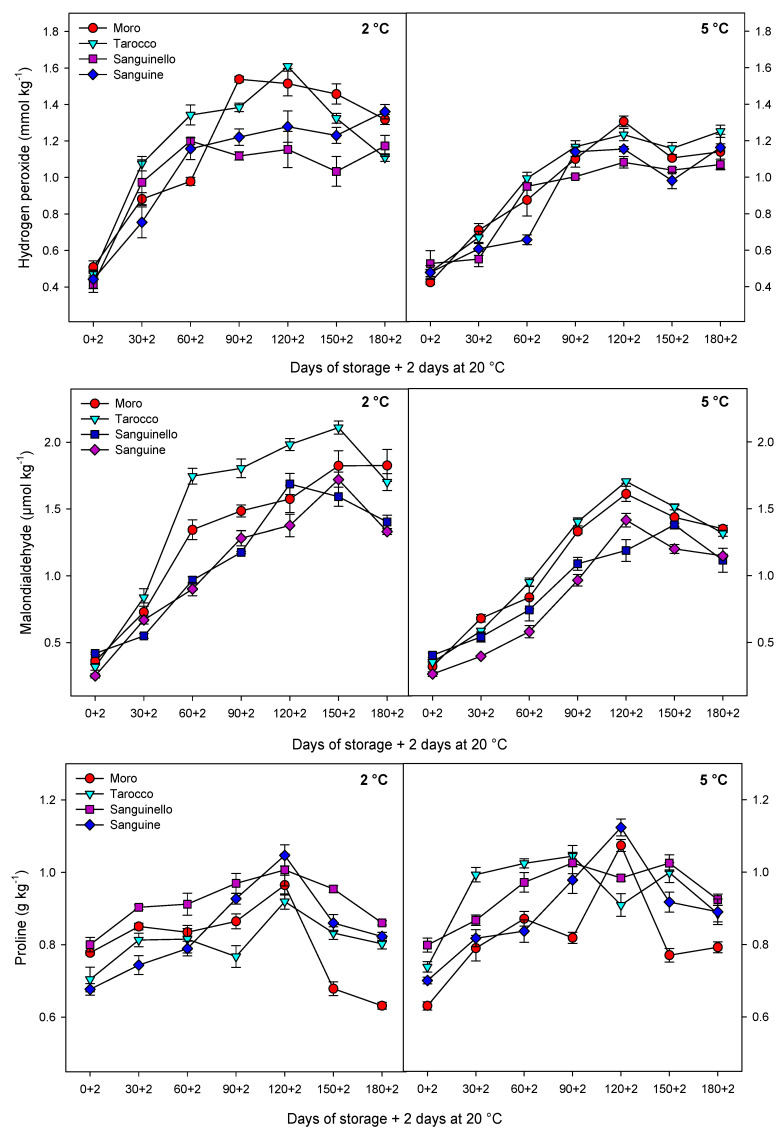
Changes in hydrogen peroxide (H_2_O_2_), malondialdehyde (MDA) and proline content in the flavedo of blood orange cultivars (‘Moro’, ‘Tarocco’, ‘Sanguinello’ and ‘Sanguine’) during cold storage at 2 and 5 °C and 90% relative humidity (RH) plus 2 days at 20 °C. Vertical bars represent ± standard error (SE) of means. LSD (*p* < 0.05) value is 0.19 for H_2_O_2_, 0.22 for MDA and 0.13 for proline.

The H_2_O_2_ accumulation was affected by cultivars, storage times and temperatures (Figure 3). The H_2_O_2_ content increased sharply in blood orange cultivars up to 120 days of cold storage. However, H_2_O_2_ content at 2 °C was 16% higher (on average) than at 5 °C. ‘Tarocco’ and ‘Moro’ had the highest H_2_O_2_ content at 2 °C (1.61 ± 0.01 and 1.51 ± 0.01 nmol kg^−1^, at 90 and 120 days, respectively) without significant differences between them. Contrarily, the lowest H_2_O_2_ content was measured in ‘Sanguinello’ and ‘Sanguine’.

There was a significant difference in MDA among cultivars at different storage times and temperatures. MDA content increased up to 150 and 120 days at 2 and 5 °C, respectively, on all cultivars, although levels were reduced in ‘Sanguine’ and ‘Sanguinello’ (Figure 3). Similarly to H_2_O_2_, the highest MDA content was measured in ‘Tarocco’ after 120 days of storage at 2 °C and also MDA content at 2 °C was 20.47% higher than at 5 °C.

As shown in Figure 3, proline content gradually increased during cold storage and then decreased. On average, proline content at 5 °C was 6.61% higher than at 2 °C. ‘Sanguinello’ had higher proline content than ‘Moro’ and ‘Tarocco’ cultivars. After 120 days of storage, the highest proline content was measured in ‘Sanguinello’ at both temperatures.

### 3.3. Antioxidant Enzyme Activities 

**Figure 4:**
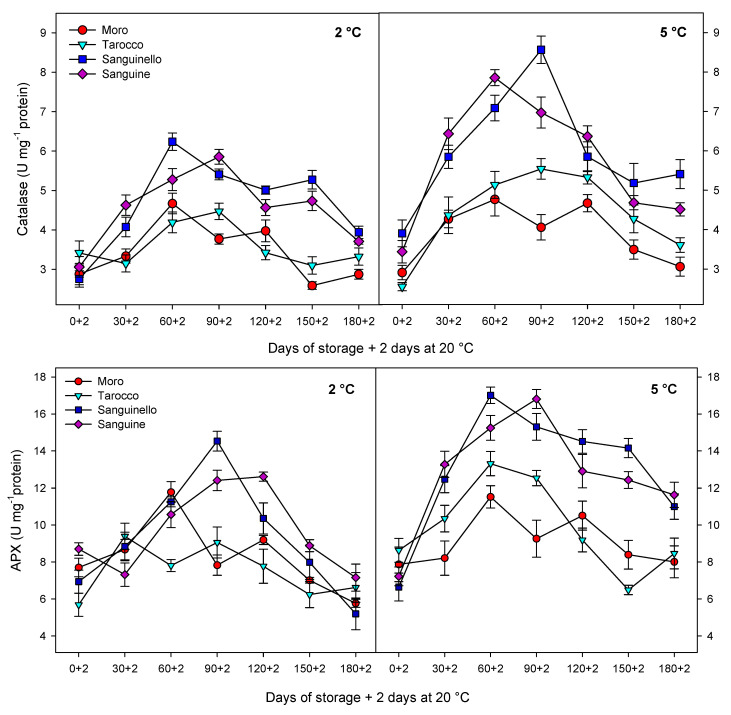
Changes in catalase (CAT) and ascorbate peroxidase (APX) enzyme activities in the flavedo of blood orange cultivars (‘Moro’, ‘Tarocco’, ‘Sanguinello’ and ‘Sanguine’) during cold storage at 2 and 5 °C and 90% relative humidity (RH) plus 2 days at 20 °C. Vertical bars represent ± standard error (SE) of means. LSD (*p* < 0.05) value is 1.10 for CAT and 2.31 for APX.

**Figure 5:**
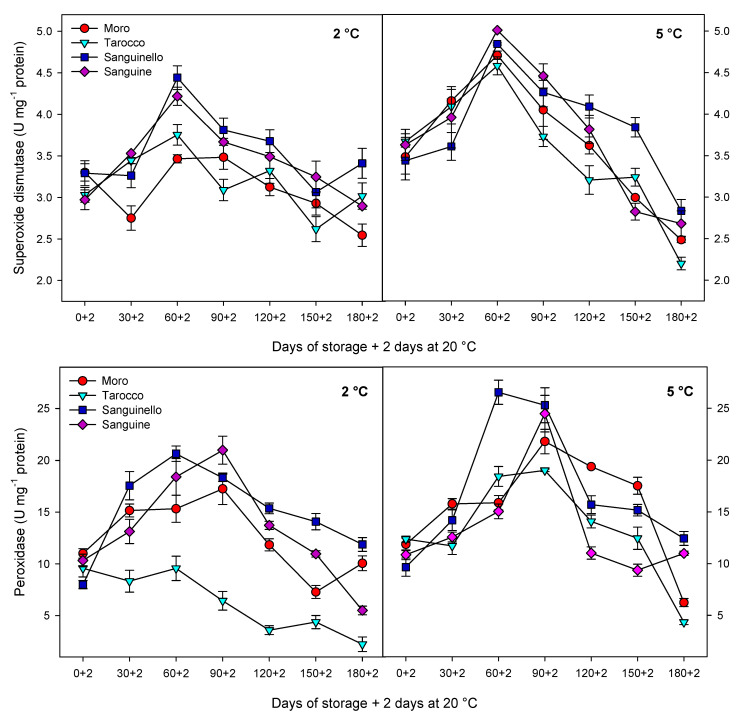
Changes in superoxide dismutase (SOD) and peroxidase (POD) enzyme activities in the flavedo of blood orange cultivars (‘Moro’, ‘Tarocco’, ‘Sanguinello’ and ‘Sanguine’) during cold storage at 2 and 5 °C and 90% relative humidity (RH) plus 2 days at 20 °C. Vertical bars represent ± standard error (SE) of means. LSD (*p* < 0.05) value is 0.61 for SOD and 3.12 for POD.

Antioxidant enzyme (CAT, APX, SOD and POD) activities in the flavedo tissue were affected by cultivars, storage times and temperatures (Figure 4 and Figure 5). CAT activity was significantly higher at 5 °C than at 2 °C for all blood orange cultivars (Figure 4). CAT activity increased up to 60 or 90 days for all cultivars at both temperatures. Among cultivars, ‘Moro’ and ‘Tarocco’ had the lowest level of CAT activity at both temperatures, whereas ‘Sanguinello’ and ‘Sanguine’ showed higher CAT activity than ‘Moro’ and ‘Tarocco’. However, there was no significant difference between ‘Sanguinello’ and ‘Sanguine’ for CAT activity. The highest CAT activity was measured in ‘Sanguinello’ after 60 days at 2 °C and after 90 days of storage at 5 °C (Figure 4). APX activity showed a significant difference among the cultivars. At both temperatures, ‘Moro’ and ‘Sanguinello’ had the lowest and the highest APX activity, respectively (Figure 4). However, on average, APX activity at 5 °C was 23% greater than at 2 °C. APX activity increased for all cultivars at days 60–90 and then decreased along the storage at both temperatures. The highest APX activity was measured in ‘Sanguinello’ after 90 days at 2 °C. 

SOD activity of cultivars was affected at both temperatures during storage (Figure 5). ‘Moro’ and ‘Tarocco’ had the lowest SOD activity at 5 °C. However, the highest SOD activity was found in ‘Sanguinello’ and ‘Sanguine’ without significant differences. As shown in Figure 5, SOD activity sharply increased for all cultivars up to 60 days of storage at 5 °C and then decreased to the end of storage. This trend was similar at 2 °C; however, SOD activity slightly increased in ‘Tarocco’ and ‘Sanguinello’ at the end of storage.

POD activity was significantly different among cultivars at both temperatures (Figure 5). POD activity at 5 °C was 20.1% higher than at 2 °C. ‘Tarocco’ had the lowest POD activity at 2 °C, whereas the highest POD activity was found in ‘Sanguinello’ after 60 days at 5 °C. However, there was no significant difference between ‘Moro’ and ‘Sanguine’ for POD activity at both temperatures.

### 3.4. PAL and PPO Enzyme Activities

**Figure 6:**
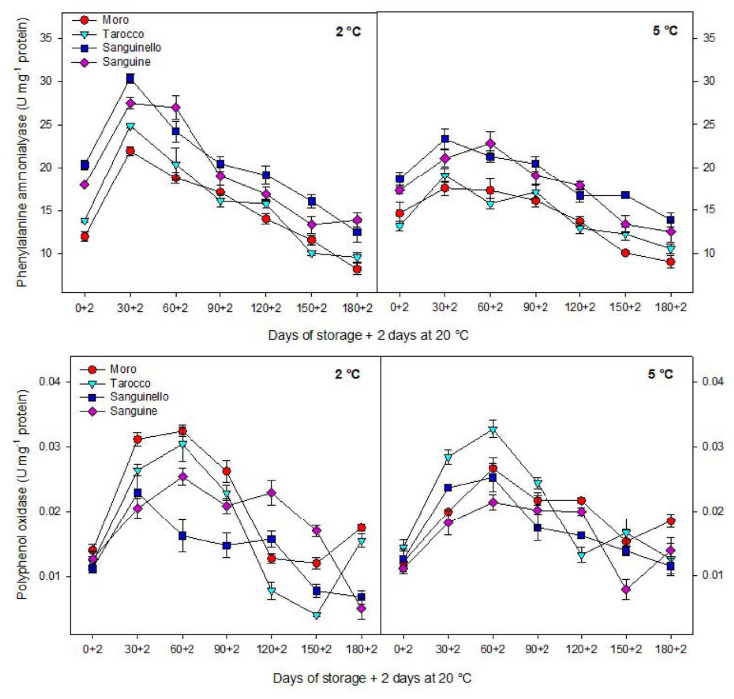
Changes in phenylalanine ammonia-lyase (PAL) and polyphenol oxidase (PPO) enzyme activities in the flavedo of blood orange cultivars (‘Moro’, ‘Tarocco’, ‘Sanguinello’ and ‘Sanguine’) during cold storage at 2 and 5 °C and 90% relative humidity (RH) plus 2 days at 20 °C. Vertical bars represent ± standard error (SE) of means. LSD (*p* < 0.05) value is 2.33 for PAL and 0.007 for PPO.

PAL activity was affected by cultivars, storage times and temperatures (Figure 6). PAL activity was significantly higher at 2 °C than at 5 °C for all blood orange cultivars. PAL activity sharply increased up to 30 days in the cultivars at 2 °C and then decreased to the end of storage. ‘Moro’ and ‘Sanguinello’ had the lowest and the highest PAL activity, respectively.

PPO activity was significantly affected by cultivars during cold storage. The highest and the lowest PPO activity were found for the ‘Moro’ and ‘Sanguinello’, respectively, at both temperatures (Figure 6). There was no significant difference between ‘Tarocco’ and ‘Sanguine’ for PPO activity. As shown in Figure 6, the activity of PPO for all cultivars increased up to 60 days and then decreased during storage. However, at the end of storage, PPO activity slightly increased in ‘Tarocco’ and ‘Sanguinello’ at 2 °C and ‘Moro’ and ‘Sanguine’ at 5 °C.

### 3.5. Hierarchical Clustering Analysis (HCA)

**Figure 7:**
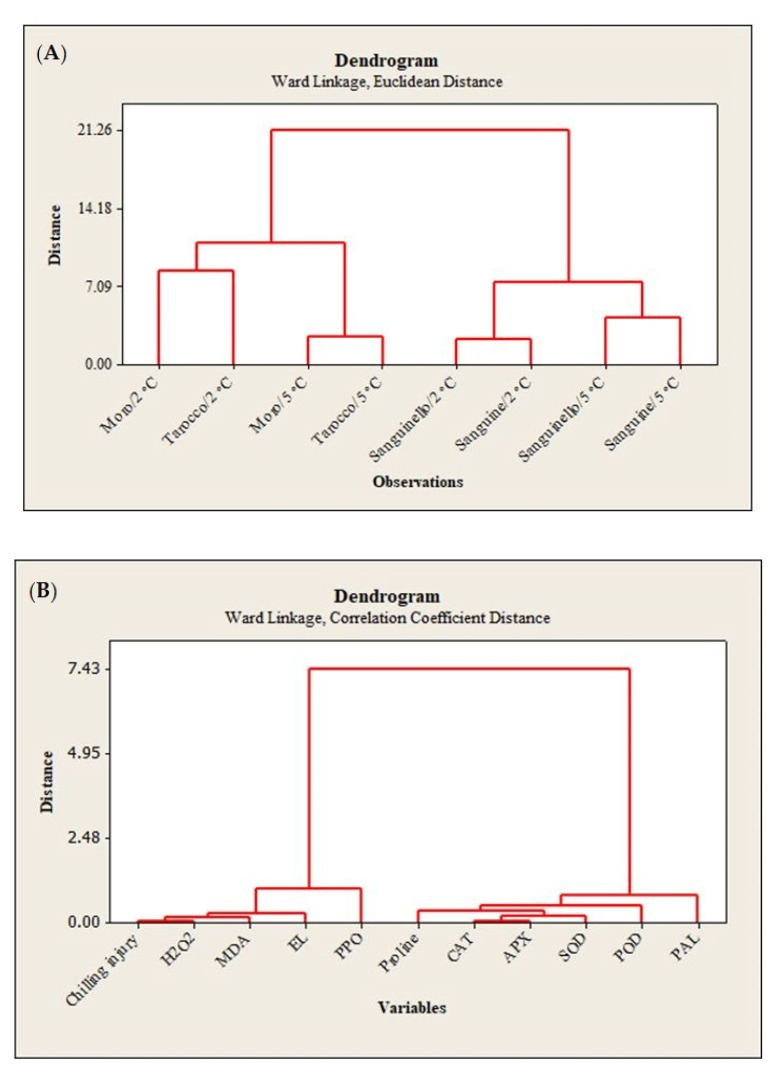
(**A**), Hierarchical clustering analysis (HCA) of the cultivars and temperatures; (**B**), parameters of blood orange cultivars (‘Moro’, ‘Tarocco’, ‘Sanguinello’ and ‘Sanguine’) during cold storage at 2 and 5 °C and 90% relative humidity (RH) plus 2 days at 20 °C.

HCA of the cultivars, temperatures and parameters is represented in Figure 7. HCA for cultivars and temperatures revealed four main clusters (Figure 7A). The first cluster included ‘Moro’ and ‘Tarocco’ at 2 °C, and the second cluster included ‘Moro’ and ‘Tarocco’ at 5 °C. The third cluster involved ‘Sanguinello’ and ‘Sanguine’ at 2 °C, and the fourth cluster included ‘Sanguinello’ and ‘Sanguine’ at 5 °C. In addition, HCA revealed two main clusters for the measured parameters (Figure 7B). CI, H_2_O_2_ and MDA contents, EL and PPO are included in the first cluster, while proline content, CAT, APX, SOD, POD and PAL were found in the second cluster.

## 4. Discussion

With regard to sensitivity of blood oranges to CI, it is essential to increase the knowledge about the fruit physiological and biochemical responses in order to identify their susceptibility to cold temperature stress. Since cold temperature can prolong the postharvest life of blood oranges, the use of low temperature is therefore unavoidable [22]. In this study, susceptibility of blood orange cultivars to CI was significantly different at both temperatures, since the occurrence of CI symptoms during storage was significantly higher at 2 °C than at 5 °C. ‘Moro’ and ‘Tarocco’ showed severe CI symptoms at 2 °C plus 2 days at 20 °C, and according to HCA (Figure 7A), ‘Moro’ and ‘Tarocco’ were close together at 2 °C or 5 °C during storage, while ‘Sanguinello’ and ‘Sanguine’ were in the same cluster at both temperatures.

Citrus fruit peel is considered the main target for the occurrence of postharvest physiological disorders, especially CI symptoms [8,23]. In this study, based on SEM micrographs, the epidermis structure of the peel of the cultivars represented the CI severity. Cold-sensitive cultivars showed higher fractures, cuticular ridges, rough surface and fissured wax crusts at low temperatures. ‘Moro’ and ‘Tarocco’ had severe fractures and cuticular ridges on the peel. On the contrary, ‘Sanguinello’ and ‘Sanguine’ showed lower fractures at both temperatures. In addition, epicuticular wax was lost at 2 °C, which demonstrated the higher CI incidence compared with 5 °C. In this study, there was a distinct difference in rind thickness among blood orange cultivars, but rind thickness of blood orange cultivars was not correlated with CI tolerance (R^2^ = 0.47 and 0.37) for 2 and 5 °C, respectively. For example, ‘Sanguine’ had a thin peel in comparison with ‘Moro’ and ‘Tarocco’, but it was a cold-tolerant cultivar. In addition, it has been reported that higher rind thickness in two citrus cultivars may be correlated with reduced postharvest peel pitting [23].

Obtained results demonstrated that, in blood oranges, the occurrence of CI symptoms is the result of a series of physiological and biochemical responses to cold temperature. The main symptoms of CI include stem-end rind breakdown, tissue softening, internal and external browning and higher susceptibility to diseases [8,24]. Primary responses to CI at the cellular level include changes in the fruit membranes and structures, which affect the membrane permeability. Low temperature affects membrane lipid phase transitions, and formation of gel phase lipids leads to increased permeability or leakiness of cellular membranes in stored fruit [25]. Additionally, responses include symptoms such as electrolyte release, decrease in metabolic energy, oxidative damage and cellular lysis [6,26]. Therefore, EL is a good indicator of membrane permeability and peroxidation of membrane fatty acids [27]. In this study, EL significantly increased for all cultivars during storage at both temperatures, but EL at 2 °C was greater than at 5 °C. ‘Sanguinello’ had the lowest EL at both temperatures. In addition, lipid peroxidation is another effect of cold stress. MDA is a secondary end product of peroxidation of membrane unsaturated fatty acid at critical temperature [7]. Furthermore, MDA is recognized as a biomarker for oxidative stress, and its product negatively correlated with cell membrane integrity [8]. In this study, cultivars had a significant difference in MDA content at both temperatures. ‘Sanguine’ and ‘Sanguinello’ had the lowest MDA content. MDA content of blood orange fruit under chilling stress significantly increased during storage and was suppressed by postharvest treatment with elicitors [8]. Based on HCA, EL and MDA content were located in the same cluster with CI (Figure 7B). In this study, EL and MDA at 2 °C were higher than at 5 °C and their amount was also significantly different among cultivars. ‘Moro’ and ‘Tarocco’ had the highest EL and MDA at 2 °C, showing that lipid peroxidation in these cultivars was more pronounced than in ‘Sanguinello’ and ‘Sanguine’.

Oxidative stress is the secondary response of susceptible fruit to CI [8]. Stored fruit at critical temperature generate ROS that subsequently damage cellular components due to oxidative stress [28]. Moreover, ROS accumulation including H_2_O_2_, singlet oxygen, superoxide and hydroxyl radicals is one of the main reasons for the incidence of CI symptoms due to oxidative damage to the cell membranes [29]. It has been reported that the H_2_O_2_ content in ‘Moro’ blood orange increased during storage at chilling temperature [8]. H_2_O_2_ can enhance membrane lipid peroxidation and oxidative damage at low temperatures. In this study, according to HCA, CI and H_2_O_2_ content were located in the same cluster (Figure 7B). H_2_O_2_ content was significantly different among cultivars at both temperatures. H_2_O_2_ accumulation at 2 °C was greater than at 5 °C, but ‘Sanguinello’ and ‘Sanguine’ had the lowest H_2_O_2_ content, which could be due to a more efficient antioxidant system. 

Antioxidant enzymes including CAT, APX and SOD play an important role in scavenging of ROS and protecting the cell membrane, subsequently decreasing CI in stored fruit at critical temperature [7]. Furthermore, enhanced levels of these enzymes have been correlated to chilling tolerance in fruit stored at critical temperature [27]. The antioxidant defence system includes a series of reactions against oxidative stress and protects components of the cell. For example, SOD is the first line of the defence system and catalyses hydrogen peroxide to superoxide anion, removes singlet oxygen and prevents formation of the hydroxide radical. Both CAT and APX decompose the hydrogen peroxide to H_2_O and O_2_ during cold stress [30]. However, CAT is considered as a key and main enzyme of the antioxidant system that is stimulated under oxidative stress caused by CI [29]. In our study, CAT, APX, SOD and POD enzyme activities were higher at 5 °C than at 2 °C for all blood orange cultivars. For example, CAT activity increased up to 60 or 90 days for all cultivars, and ‘Moro’ and ‘Tarocco’ had the lowest CAT activity at both temperatures. APX activity increased at days 60–90 and then decreased for all cultivars at both temperatures. ‘Moro’ and ‘Tarocco’ had the lowest SOD activity. This result may be due to injury to cell membrane components, in turn inducing the transition and formation of the flexible crystal–liquid form to a solid–gel phase at low temperatures [25]. Therefore, the main reason for the occurrence of CI symptoms in these cultivars was probably ROS overproduction due to the reduction of antioxidant activities [8]. A previous study demonstrated in ‘Moro’ blood oranges the efficiency of elicitor treatment that enhanced chilling tolerance by increasing the antioxidant enzyme activity at cold stress [8]. In ‘Eureka’ lemon fruit, total antioxidant capacity decreased during cold storage period [31]. It has been reported that the antioxidant profile and the antioxidant activity of five sweet orange genotypes were affected during cold storage at 6 ± 1 °C for 65 days [32]. In this study, among the cultivars, ‘Sanguinello’ and ‘Sanguine’ had the highest levels of antioxidant enzyme activities at both temperatures. In addition, the lowest activity of antioxidant enzymes was found in ‘Moro’ and ‘Tarocco’, which had the highest susceptibility to CI. Therefore, higher antioxidant enzyme activities in blood orange cultivars significantly eliminated H_2_O_2_ content and reduced the incidence of CI. Antioxidant enzymes in all cultivars increased up to 60–90 days during storage and then decreased, which might be due to the reduction of energy status or fruit senescence leading to the production of H_2_O_2_ that predominated over the antioxidant enzymes’ scavenging activity [8]. 

Proline has a contribution to integrity and stability of cellular membranes, membrane structures, ROS scavenging and protein protection and balances the cytoplasmic osmotic potential, leading to increased fruit resistance to CI [33]. Furthermore, proline accumulation is an adaptive mechanism to help fruit cells against damage from oxidative stress during low temperature storage [8]. Recently, research has shown that proline accumulation can enhance chilling tolerance in stored fruit at critical temperatures [7,8]. In accordance with this, proline is positively correlated with chilling tolerance in blood orange fruit [8]. In our study, the lowest and highest proline content were observed in ‘Moro’ and ‘Sanguinello’, respectively, whereas there was no significant difference between ‘Tarocco’ and ‘Sanguine’. Therefore, proline could maintain membrane integrity through osmoregulation at chilling temperature [7].

PAL activity has been considered as a defence mechanism of cold stored fruit in response to chilling stress [8,28,33]. Cold temperature can enhance PAL activity in blood orange by upregulating PAL codifying genes in response to chilling stress [9]. In this sense, PAL mRNA accumulated in mandarin fruit during storage at low temperature (2 °C), which was related to the sensitivity of citrus fruit to CI [34]. In addition, induction of PAL is required for producing some metabolites that can help the cold stored fruit to resist chilling stress [35]. In this study, PAL activity was significantly different in blood orange cultivars and storage temperatures. ‘Sanguinello’ and ‘Moro’ had the highest and the lowest PAL activity, respectively, during storage. In addition, PAL activity at 2 °C was higher than at 5 °C for all blood orange cultivars. PAL activity is a good biochemical marker for evaluating chilling tolerance in fruit during low temperature storage since enhancement of PAL activity was associated with increased chilling tolerance in blood orange fruit. Furthermore, PAL activity is important to reduce chilling symptoms and postharvest physiological disorders of fruit exposure to cold stress [36]. It has been reported that long storage periods at 4 °C impacted on the expression of structural genes involved in anthocyanin biosynthesis such as PAL [37]. In the present study, PAL enzyme was correlated with chilling tolerance of blood orange cultivars according to HCA. However, PAL activity sharply increased up to 60 days and then decreased to the end of storage. This evidence permits the inference that the increase in PAL at the initial occurrence of CI symptoms may reveal the initiation of cellular component damage. Therefore, severity of CI in blood orange cultivars would be dependent on the intensity of cell damage associated with the potential of physiological and biochemical responses at cold stress. This event probably is due to the reduction in activity of antioxidant enzymes, fruit senescence as well as the reduction of cellular energy that induced CI symptoms in blood orange cultivars [8].

In this study, HCA showed that CI in blood orange cultivars could also be attributed to PPO activity (Figure 7B). The lowest PPO activity was found in ‘Sanguinello’ at both temperatures. PPO activity increased up to 60 days in all cultivars and then decreased to the end of storage. PPO is responsible for oxidation of phenols to highly toxic quinones during cold stress. In addition, PPO activity can oxidize antioxidants, subsequently decreasing the antioxidant activity of cold stored fruit and leading to the induction of CI and loss of membrane integrity during cold storage [8]. Therefore, low PPO activity appears to be related to a lower CI symptom in ‘Sanguinello’ cultivar. On the contrary, the cold sensitivity of ‘Moro’ is associated with the lower PAL and higher PPO enzyme activities.

In blood oranges, chilling stress can decrease the fruit quality and bioactive compounds during cold storage [22]. Moreover, chilling injury observed on the rind is related to fruit quality and marketability of blood orange fruit. Our previous publication demonstrated that the tolerance cultivar had a higher quality than the susceptible cultivar as it did in ‘Sanguinello’ [38].

## 5. Conclusions

This is the first comprehensive study for chilling tolerance of blood orange cultivars at low temperatures. Rind thickness of blood orange cultivars was not correlated with chilling tolerance. According to HCA, H_2_O_2_, MDA, EL and PPO were located in the same cluster with CI. On the contrary, proline content, antioxidant activity and PAL were correlated for enhancing chilling tolerance. The highest capacity of antioxidant enzymes prevented the loss of membrane integrity and permeability, and subsequently decreased the incidence of CI at critical temperature. On the basis of SEM micrographs, epidermis structure had severe fractures and cuticular ridges on the peel of cold sensitive cultivars. ‘Sanguinello’ and ‘Sanguine’ stored at low temperatures were more tolerant to chilling stress, probably due to more efficient antioxidant enzyme activities and higher PAL and lower PPO than those of sensitive cultivars. On the basis of these findings, the order of susceptibility of cultivars to CI was ‘Moro’ > ‘Tarocco’ > ‘Sanguine’ > ‘Sanguinello’.

## References

[1] Habibi F., Ramezanian A. (2017). Vacuum infiltration of putrescine enhances bioactive compounds and maintains quality of blood orange during cold storage. Food Chem..

[2] Molinu M.G., Dore A., Palma A., D’Aquino S., Azara E., Rodov V., D’Hallewin G. (2016). Effect of superatmospheric oxygen storage on the content of phytonutrients in ‘Sanguinello Comune’ blood orange. Postharvest Biol. Technol..

[3] Butelli E., Licciardello C., Zhang Y., Liu J., Mackay S., Bailey P., Reforgiato-Recupero G., Martin C. (2012). Retrotransposons Control Fruit-Specific, Cold-Dependent Accumulation of Anthocyanins in Blood Oranges. Plant Cell.

[4] Fallico B., Ballistreri G., Arena E., Brighina S., Rapisarda P. (2017). Bioactive compounds in blood oranges (*Citrus sinensis* (L.) Osbeck): Level and intake. Food Chem..

[5] Hodgson R.W., Reuther W., Webber H.J., Batchelor L.D. (1967). Horticultural varieties of Citrus. The Citrus Industry.

[6] Yang A., Cao S., Yang Z., Cai Y., Zheng Y. (2011). γ-Aminobutyric acid treatment reduces chilling injury and activates the defence response of peach fruit. Food Chem..

[7] Wang Y., Luo Z., Huang X., Yang K., Gao S., Du R. (2014). Effect of exogenous γ-aminobutyric acid (GABA) treatment on chilling injury and antioxidant capacity in banana peel. Sci. Hortic..

[8] Habibi F., Ramezanian A., Rahemi M., Eshghi S., Guillén F., Serrano M., Valero D. (2019). Postharvest treatments with γ -aminobutyric acid, methyl jasmonate, or methyl salicylate enhance chilling tolerance of blood orange fruit at prolonged cold storage. J. Sci. Food Agric..

[9] Rehman M., Singh Z., Khurshid T. (2018). Alleviation of chilling injury induced by cold quarantine treatment in Midknight Valencia and Lane Late sweet orange fruit. Aust. J. Crop. Sci..

[10] Schirra M., Mulas M., Fadda A., Cauli E. (2004). Cold quarantine responses of blood oranges to postharvest hot water and hot air treatments. Postharvest Biol. Technol..

[11] Rapisarda P., Bellomo S.E., Intelisano S. (2001). Storage Temperature Effects on Blood Orange Fruit Quality. J. Agric. Food Chem..

[12] Palma A., D’Aquino S., Vanadia S., Angioni A., Schirra M. (2013). Cold quarantine responses of ‘Tarocco’ oranges to short hot water and thiabendazole postharvest dip treatments. Postharvest Biol. Technol..

[13] Nukuntornprakit O.-A., Chanjirakul K., Van Doorn W.G., Siriphanich J. (2015). Chilling injury in pineapple fruit: Fatty acid composition and antioxidant metabolism. Postharvest Biol. Technol..

[14] Shang H., Cao S., Yang Z., Cai Y., Zheng Y. (2011). Effect of Exogenous γ-Aminobutyric Acid Treatment on Proline Accumulation and Chilling Injury in Peach Fruit after Long-Term Cold Storage. J. Agric. Food Chem..

[15] Bradford M.M. (1976). A rapid and sensitive method for the quantitation of microgram quantities of protein utilizing the principle of protein-Dye binding. Anal. Biochem..

[16] Chance B., Maehly A. (1955). Assay of catalases and peroxidases. Methods Enzymol..

[17] Nakano Y., Asada K. (1981). Hydrogen Peroxide is Scavenged by Ascorbate-specific Peroxidase in Spinach Chloroplasts. Plant Cell Physiol..

[18] Beauchamp C., Fridovich I. (1971). Superoxide dismutase: Improved assays and an assay applicable to acrylamide gels. Anal. Biochem..

[19] Liu Q., Xi Z., Gao J., Meng Y., Lin S., Zhang Z. (2016). Effects of exogenous 24-epibrassinolide to control grey mould and maintain postharvest quality of table grapes. Int. J. Food Sci. Technol..

[20] Da Silva C.R., Koblitz M.G.B. (2010). Partial characterization and inactivation of peroxidases and polyphenol-oxidases of umbu-cajá (*Spondias* spp.). Food Sci. Technol..

[21] Khorram F., Ramezanian A., Hosseini S.M.H. (2017). Shellac, gelatin and Persian gum as alternative coating for orange fruit. Sci. Hortic..

[22] Habibi F., Ramezanian A., Guillén F., Serrano M., Valero D. (2020). Blood oranges maintain bioactive compounds and nutritional quality by postharvest treatments with γ-aminobutyric acid, methyl jasmonate or methyl salicylate during cold storage. Food Chem..

[23] Cronjé P.J., Zacarías L., Alférez F. (2017). Susceptibility to postharvest peel pitting in Citrus fruits as related to albedo thickness, water loss and phospholipase activity. Postharvest Biol. Technol..

[24] Lafuente M.T. (2006). Postharvest physiological disorders in citrus fruit. Stewart Postharvest Rev..

[25] Cao S., Yang Z., Cai Y., Zheng Y. (2011). Fatty acid composition and antioxidant system in relation to susceptibility of loquat fruit to chilling injury. Food Chem..

[26] Valero D., Serrano M. (2010). Postharvest Biology and Technology for Preserving Fruit Quality.

[27] Jin P., Zhu H., Wang J., Chen J., Wang X., Zheng Y. (2012). Effect of methyl jasmonate on energy metabolism in peach fruit during chilling stress. J. Sci. Food Agric..

[28] Siboza X.I., Bertling I., Odindo A.O. (2017). Enzymatic antioxidants in response to methyl jasmonate and salicylic acid and their effect on chilling tolerance in lemon fruit [*Citrus limon* (L.) Burm. F.]. Sci. Hortic..

[29] Sevillano L., Sanchez-Ballesta M.T., Romojaro F., Flores F.B. (2009). Physiological, hormonal and molecular mechanisms regulating chilling injury in horticultural species. Postharvest technologies applied to reduce its impact. J. Sci. Food Agric..

[30] Huang R.-H., Liu J.-H., Lu Y.-M., Xia R. (2008). Effect of salicylic acid on the antioxidant system in the pulp of ‘Cara cara’ navel orange (*Citrus sinensis* L. Osbeck) at different storage temperatures. Postharvest Biol. Technol..

[31] Sun Y., Singh Z., Tokala V.Y., Heather B. (2019). Harvest maturity stage and cold storage period influence lemon fruit quality. Sci. Hortic..

[32] Rapisarda P., Bianco M.L., Pannuzzo P., Timpanaro N. (2008). Effect of cold storage on vitamin C, phenolics and antioxidant activity of five orange genotypes [*Citrus sinensis* (L.) Osbeck]. Postharvest Biol. Technol..

[33] Sun H., Luo M., Zhou X., Zhou Q., Sun Y., Ge W., Wei B., Cheng S., Ji S.-J. (2020). Exogenous glycine betaine treatment alleviates low temperature-induced pericarp browning of ‘Nanguo’ pears by regulating antioxidant enzymes and proline metabolism. Food Chem..

[34] Sanchez-Ballesta M.T., Zacarías L., Granell A., Lafuente M.T. (2000). Accumulation of PAL transcript and PAL activity as affected by heat-conditioning and low-temperature storage and its relation to chilling sensitivity in mandarin fruits. J. Agric. Food Chem..

[35] Sayyari M., Babalar M., Kalantari S., Serrano M., Valero D. (2009). Effect of salicylic acid treatment on reducing chilling injury in stored pomegranates. Postharvest Biol. Technol..

[36] Lafuente M.T., Zacarías L., Martínez-Téllez M.A., Sanchez-Ballesta M.T., Granell A., Lafuente M.T. (2003). Phenylalanine ammonia-lyase and ethylene in relation to chilling injury as affected by fruit age in citrus. Postharvest Biol. Technol..

[37] Piero A.R.L., Puglisi I., Rapisarda A.P., Petrone G. (2005). Anthocyanins Accumulation and Related Gene Expression in Red Orange Fruit Induced by Low Temperature Storage. J. Agric. Food Chem..

[38] Habibi F., Ramezanian A., Guillén F., Castillo S., Serrano M., Valero D. (2020). Changes in Bioactive Compounds, Antioxidant Activity, and Nutritional Quality of Blood Orange Cultivars at Different Storage Temperatures. Antioxidants.

